# Anti-aging potential of *Monascus purpureus* pigments in skin care

**DOI:** 10.1080/13880209.2025.2557983

**Published:** 2025-09-18

**Authors:** Rittipun Rungruang, Tasanee Panichakul, Napassorn Peasura, Sasithorn Kongruang

**Affiliations:** a Department of Cosmetic Science, Faculty of Science and Technology, Suan Dusit University, Bangkok, Thailand; b Applied Microbiology, Institute of Food Research and Product Development, Kasetsart University, Bangkok, Thailand; c Department of Biotechnology, Faculty of Applied Science, King Mongkut’s University of Technology North Bangkok, Bangkok, Thailand

**Keywords:** *Monascus* pigments, collagen and elastin production, wound healing, anti-collagenase, anti-elastase, natural pigments, fermentation

## Abstract

**Context:**

Natural pigments derived from microbial sources are garnering increasing attention owing to their multifunctional roles in cosmetic and biomedical applications.

**Objective:**

We investigated the antioxidant and biological activities of biocolor extracts of *Monascus purpureus* cultivated in potato dextrose broth supplemented with 1% Tubtim chumphae (*Oryza sativa* L., Poaceae) broken rice (MPTR).

**Materials and methods:**

*M. purpureus* TISTR 3615 pigment yield and quality under fermentation conditions with and without 1% Tubtim chumphae broken rice were examined. Liquid chromatography was performed to determine and quantify the key components of the extracts. The biological activities of the extracts were investigated using antioxidant and enzyme inhibition assays. Cell viability assay was performed to determine the safety of the extracts on human dermal fibroblasts. Furthermore, cellular collagen and elastin production promoted by the extracts was also investigated.

**Results:**

Notably, MPTR extract demonstrated superior pigment production compared with conventional potato dextrose broth cultured fungi. MPTR extract exhibited potent antioxidant properties and was non-cytotoxic to human skin fibroblasts at concentrations up to 0.5 mg/mL. MPTR extract effectively inhibited collagenase and elastase enzymes, and induced cellular collagen type I and elastin production. Moreover, MPTR extract promoted wound healing, significantly reducing the wound area by up to 90% within 48 h.

**Discussion and conclusion:**

MPTR extract from *M. purpureus* TISTR 3615 fermented with broken rice exhibited antioxidant properties as well as collagenase and elastase inhibitory activities, and promoted cellular collagen type I and elastin production. MPTR extract is a promising skin anti-aging agent for cosmetic and cosmeceutical applications.

## Introduction

The extracellular matrix (ECM), composed of collagen, elastin, and hyaluronic acid, is responsible for maintaining skin elasticity and strength. Aging, which is driven by intrinsic and extrinsic factors, reduces ECM production, leading to wrinkles and sagging. Notably, free radicals and inflammation accelerate skin aging by activating collagenases and elastases, which degrade collagen and elastin (Mukherjee et al. 2011; Sin and Kim 2005). The demand for natural bioactive ingredients derived from plants, fungi, and microbes capable of counteracting aging and promoting skin health has recently increased (Wijesekara and Xu 2023; Essmat et al. 2024). Biocolors are natural colorants derived from biological sources, including plants, insects, and microbes (Singh et al. 2023). Pigments produced by bacteria and fungi play crucial roles in cell survival and cytology. Importantly, these pigments can inhibit bacterial growth at low concentrations and possess various health benefits in humans, including antioxidant, anticancer, and anti-inflammatory properties (Chaudhary et al. 2022; Husakova and Patakova 2025). Additionally, fungal pigments effectively protect tissues such as blood, breast, liver, and skin (Banach et al. 2022; Adin et al. 2023). Fungal pigments are gaining popularity because of their natural and nontoxic nature, rapid growth, and independence from weather conditions, making them suitable for large-scale production (Usmani et al. 2020; Cavalcante et al. 2023). Fungal pigments have been applied in both medical and agricultural research. *Monascus purpureus,* a red mold species, produces natural pigments that can be used as food colorants and additives (Abdel-Raheam et al. 2022; Arruda et al. 2025). Pigments synthesized by fatty acid and polyketide synthases include yellow, orange, and red pigments (Shu et al. 2022). Recent reviews highlight further biological functionalities of *Monascus* pigments, including antioxidant, anti-inflammatory, neurocytoprotective, anti-obesity, and hepatoprotective properties (Egea et al. 2023). A systematic analysis of specific *Monascus* pigments such as ankaflavin, monacorubrin, monascin, and rubropunctamine demonstrated potent antioxidant activity, with monacorubrin showing particularly strong radical-scavenging capacity in chemical assays (Chen et al. 2025). However, *M. purpureus* also produces mycotoxins (citrinin) that are harmful to the liver and kidneys (Silva et al. 2020). Recently, citrinin-free strain *M. purpureus* BCRC 38110 has been established and isolated compounds from this strain exhibited dermal protective activities (Wu et al. 2021a). Research findings indicate that *Monascus* efficiently produces pigments through fermentation, utilizing agricultural by-products such as cassava pulp (Jirasatid and Nopharatana 2016), rice bran (Jirasatid and Nopharatana 2017), coffee pulp (Jirasatid and Nopharatana 2016), corn cob (Nimnoi and Lumyong 2011), jackfruit seed powder (Babitha et al. 2006), corn stalks (Velmurugan et al. 2011), and durian seed powder (Srianta et al. 2012). Among these, rice bran has garnered attention because of its high nutritional value and characteristics as an excellent source of carbohydrates, proteins, and essential minerals. For example, *M. ruber* effectively produced red pigments from broken rice in a solid-state fermentation, reducing production cost and serving as a sustainable source of red pigments (Gomah et al. 2017). *Monascus* pigments contain pharmaceutical compounds with anti-inflammatory, antidiabetic, and enzyme-inhibitory properties (Gong et al. 2023; Husakova and Patakova 2025; Liu et al. 2025). Jin and Pyo (2017) reported that an extract from soybean fermentation with *M. pilosus* KCCM 60084 exhibited antioxidant properties and inhibitory activities against skin aging-related enzymes, including tyrosinase, hyaluronidase, and elastase. Additionally, Koli et al. (2019) utilized *M. purpureus* extract as an additive in commercial sunscreens, highlighting its potential for enhancing sun protection. Furthermore, (Wu et al. 2021a; Wu et al. 2021b) investigated the chemical components of *M. purpureus* with photoprotective and anti-melanogenic activities, providing further evidence of its skin-protective properties. Research findings indicate that pigments, such as rubropunctamine (red pigment), monascin, and ankaflavin (yellow pigment), exhibit antioxidant properties and enhance the sun protection factor in sunscreen (Lebeau et al. 2017; Koli et al. 2019). Moreover, pigments derived from *Monascus* have antioxidant activity and are non-cytotoxic to human keratinocytes and erythrocytes (Koli et al. 2019). These pigments show strong potential for incorporation into cosmetic formulations, including facial creams, lotions, sunscreens, and anti-aging creams, due to their diverse color shades, water solubility, and beneficial bioactivities.

Therefore, we investigated the antioxidant and biological activities of biocolor extracts of *M. purpureus* cultivated in potato dextrose broth supplemented with 1% Tubtim chumphae (*Oryza sativa* L., Poaceae) broken rice (MPTR). The antioxidant capacity, collagenase and elastase inhibitory activities, cytotoxicity, elastin- and collagen-stimulating properties, and wound healing of MPTR extract were examined. Overall, this extract could be utilized in the development of cosmeceuticals and color cosmetics.

## Materials and methods

### Fermentation and extraction of M. purpureus TISTR 3615

*M. purpureus* TISTR 3615 was obtained from the Thailand Institute of Scientific and Technological Research (Bangkok, Thailand). The rice sample of *Oryza sativa* L. (Poaceae) (Tubtim chumphae variety, Batch No. KM069) was kindly provided by the Department of Rice, Ministry of Agriculture and Cooperatives, Thailand, and originally cultivated in Sai Ngam District, Kamphaeng Phet Province, was broken and used as a nutritional source to enhance pigment production by *M. purpureus*. The fungal strain was initially cultured in potato dextrose broth (PDB) at 30 °C for 2 days and transferred to potato dextrose agar (PDA) for 7 days. Thereafter, the fungal mycelia were harvested using a 0.5-cm diameter cork borer, inoculated into 50 mL of PDB with or without 1% Tubtim chumphae broken rice (Rungruang et al. 2019) in a 250 mL flask, and cultured in a shaking incubator at 200 rpm for 3 and 7 days at 30 °C. Mycelial morphology and *Monascus* pigments were observed microscopically. For the fermentation, 500 mL of spore culture with a spore concentration of 2.90 × 10^6^ spores/mL was inoculated into a 5-L bioreactor (BioFlo 120, Eppendorf, New York, MA, USA). The growth condition was controlled at 30 °C and pH of 7–8, under constant stirring at 100 rpm and aeration at a rate of 1 L/min, with oxygen dissolution > 30% (Kongruang 2011). Samples of *M. purpureus* cultivated in PDB (MP) and *M. purpureus* cultivated in PDB with 1% broken rice (MPTR) were collected at 7 days of fermentation.

The collected samples were filtered and extracted with 95% ethanol for 24 h. After removing the alcohol using a rotary evaporator, the extracts were stored at –20 °C for subsequent freeze-drying using a freeze dryer (LABCONCO, USA). The dried extracts were weighed to determine the percentage yield. The pigment content and color properties were measured by the absorbance at 400, 460, and 500 nm for yellow, orange, and red pigments, respectively, using a microplate reader (Biochrom EZ Read 2000, Cambridge, United Kingdom). The pigment content was expressed as absorbance units (AU/mL).

### Characterization of M. purpureus extracts by liquid chromatography-mass spectrometry (LC–MS)

Briefly, the dried extract (1 mg) was dissolved in methanol, homogenized using an ultrasonic homogenizer for 30 min, centrifuged at 959 *g* for 15 min to collect the supernatant, which was transferred to a vial. Notably, the extraction process was repeated four times by adding 2 mL of methanol. All supernatants were combined to a total volume of 10 mL and analyzed by LC-MS. Separation was performed using an Intersil ODS-3 column (4.6 × 150 mm, 5 µm) with a guard column (4.0 × 10 mm, 5 µm) at 40 °C. The mobile phases were (A) DI water with 0.1% formic acid and (B) acetonitrile with 0.1% formic acid, with a gradient program of 40% B at 0 min, 100% B at 25 min, returning to 40% B at 25.10 min, and held for 10 min. The flow rate was 0.2 mL/min, injection volume was 5 µL, and UV detection was monitored at 400 and 500 nm. LC–MS data were acquired on an LCMS-IT-TOF (Shimadzu) using electrospray ionization (ESI) in both positive and negative modes. The MS acquisition included a full-scan survey (m/z 100–1000) in both modes, followed by targeted MS/MS in positive mode for selected precursor ions at a fixed collision energy (40 eV). Compound identification was based on accurate mass (±5 ppm), retention time, UV–Vis maxima, and characteristic MS/MS fragments, compared with literature data (Avula et al. 2015).

### Determination of antioxidant properties

#### 2,2-Diphenyl-1-picrylhydrazyl (DPPH•) radical-scavenging assay

Briefly, the antioxidant activity of *M. purpureus* pigments was determined using DPPH radical scavenging assay (Rungruang et al. 2021). Specifically, DPPH was purchased from Sigma-Aldrich (St. Louis, MO, USA; Catalogue No. D9132) and prepared as a 0.16 mM solution in 95% ethanol (195 µL) was mixed with the extracts (5 µL) in a 96-well plate and kept in the dark for 30 min. Finally, the absorbance at 515 nm was measured using a microplate reader, and the scavenging activity of the extract was calculated and expressed as the IC_50_ value (the concentration of extract capable of scavenging 50% of DPPH radicals).

#### 2,2-Azino-bis-(3-ethylbenzo-thiazoline-6-sulfonic acid) (ABTS•+) radical-scavenging assay

ABTS was purchased from Sigma-Aldrich (St. Louis, MO, USA; Catalogue No. A1888) and used to generate ABTS radical, 7.0 mM ABTS and 2.45 mM K_2_S_2_O_8_ were mixed as previously described by Rungruang et al. (2021), followed by incubation in the dark for 16 h. Thereafter, the mixture was diluted with 95% ethanol, and the absorbance was measured at 734 nm until an accurate reading of 0.70 ± 0.05 U was obtained. Finally, the free radical solution (50 µL) was mixed with the extract (150 µL) in a 96-well plate and kept in the dark for 6 min. ABTS radical-scavenging activity was determined by measuring the optical density (OD) at 734 nm, and expressed as the IC_50_ value.

#### Ferric reducing antioxidant power (FRAP) assay

FRAP assay was performed to assess the antioxidant effect of the extract following a modified procedure described by Wojtunik-Kulesza (2020). Briefly, the FRAP reagent was freshly prepared by mixing 300 mM acetate buffer (pH 3.6), 10 mM 2,4,6-tri(2-pyridyl)-S-triazine (TPTZ; Sigma-Aldrich (St. Louis, MO, USA; Catalogue No. T0672) dissolved in 40 mM HCl, and 20 mM iron (III) chloride solution in a 10:1:1 ratio (v/v/v). Thereafter, the extract (20 µL) was mixed with 150 µL of FRAP reagent and incubated at 37 °C for 8 min. Absorbance was measured at 593 nm using a microplate reader. Finally, the FRAP value was calculated based on a Trolox standard curve (0–100 µM) and expressed as Trolox equivalents (TE) per gram of extract.

### Enzyme inhibitory assays

#### Anti-collagenase activity assay

Briefly, the collagenase inhibitory activity of the extract was assessed using EnzChek® Gelatinase/Collagenase Assay Kit (Molecular Probes, Eugene, OR, USA; Catalogue No. E12055), following the fluorometric method described by Rungruang et al. (2021). Specifically, 20 µL of the extract and 100 µL of collagenase from *Clostridium histolyticum* were mixed with 20 µL of DQ™ collagen type I substrate in a 96-well black plate and incubated in the dark at 25 °C for 90 min. Thereafter, the fluorescence intensity was measured using a fluorescence microplate reader (Infinite® 200 PRO, Switzerland) at excitation and emission wavelengths of 485 and 538 nm, respectively, with epigallocatechin gallate (EGCG) as the positive control.

#### Anti-elastase activity assay

The elastase inhibitory activity of the extract was evaluated following the method described by Rungruang et al. (2021). Briefly, 10 µL of each extract solution (1 mg/mL) was mixed with 4.5 unit/mL pancreatic elastase enzyme, PE-E.C.3.4.21.36 (Sigma-Aldrich, St. Louis, MO, USA; Catalogue No. E0258) and incubated at room temperature for 15 min. Thereafter, 1.6 mM N-succinyl-Ala-Ala-Ala-p-nitroanilide (AAAPVN; Sigma-Aldrich, St. Louis, MO, USA; Catalogue No. S4760) solution and 0.2 mM Tris–HCl buffer (pH 8.0) were added. Finally, the absorbance was measured at 410 nm using a microplate reader (Infinite® 200 PRO, Switzerland), with epigallocatechin gallate (EGCG) as the positive control.

### Cytotoxic assay

WST-assay was performed to investigate the potential cytotoxicity of the extract to human skin fibroblasts as described by Tan and Berridge (2000). Briefly, human neonatal dermal fibroblasts (HDFn; Homo sapiens, CVCL: 9490) were obtained from the American Type Culture Collection (ATCC, Manassas, VA, USA; Catalogue No. PCS-201-010). The cells were seeded in a 96-well plate (2 × 10^4^ cells/well) containing Dulbecco’s modified Eagle’s medium (DMEM; Gibco™, Thermo Fisher Scientific, Catalogue No. 11965-092) supplemented with 10% fetal bovine serum (FBS; Gibco™, Thermo Fisher Scientific, Catalogue No. 10270-106, qualified, origin: USA) and 1% penicillin/streptomycin (Pen/Strep; Gibco™, Thermo Fisher Scientific, Catalogue No. 15140-122). Thereafter, the plate was incubated at 37 °C with 5% CO_2_ for 24 h, followed by the addition of various concentrations (0.01, 0.03, 0.06, 0.12, 0.25, 0.50, 1, 2, 5, and 10 mg/mL) of MPTR extract and further incubated for 24 h. After washing with phosphate buffer, WST-1 solution (10 µL/well) and fresh media (100 µL/well) were added to the wells, followed by incubation for 30 min. At the end of the incubation period, absorbance was measured at 450 nm.

### Evaluation of cellular collagen type I and elastin production

HDFn cells (1 × 10^5^ cells/well) were seeded in 24-well plates and cultured in DMEM supplemented with 10% FBS for 24 h, followed by treatment with various concentrations of MPTR extract (0.125, 0.25, and 0.5 mg/mL) for 24 h. DMEM and L-ascorbic acid (30 µg/mL) were used as the negative and positive controls, respectively. Finally, the culture supernatant was harvested to detect collagen, and the treated cells were collected to determine elastin levels.

#### Determination of cellular collagen type I by ELISA kit

Briefly, the effect of the extract on collagen production was determined using a Human Collagen Type I ELISA kit (Cosmo Bio, USA, Catalogue No. ACE-EC1-E105), following the method described by Morakul et al. (2024). Specifically, 50 µL of a mixture of cell culture supernatant and biotinylated anti-collagen antibody in the ratio 1:9 was added in 96-well plate coated with polyclonal antibody to human type 1 collagen and incubated at room temperature for 1 h. After three washes with wash buffer, avidin-HRP conjugate solution was added, followed by incubation at room temperature for 1 h. Thereafter, the samples were washed three times with wash buffer, and the reaction was initiated by adding TMB substrate solution. After incubating for 15 min, the reaction was stopped, and the absorbance was measured at 450 nm. The concentration of Type I collagen was estimated using a linear regression graph of a collagen standard.

#### Detection of cellular elastin by Fastin™ elastin assay

Briefly, the elastin stimulatory effect of the extract was assessed using the elastin assay – Fastin^TM^ kit (Biocolor, UK, Catalogue No. F2000), as described by Siquier-Dameto et al. (2023). Specifically, elastin was extracted from the cell pellets using 0.25 M oxalic acid, followed by heating at 100 °C for 1 h and centrifugation at 10,000 rpm for 10 min at 4 °C (two times). Thereafter, the supernatant was precipitated with an equal volume of a precipitating reagent, followed by incubation for 15 min and centrifugation of the supernatant at 10,000 rpm for 10 min at 4 °C to collect pellets containing elastin. Finally, the dye dissociation reagent (250 µL) was added and the absorbance was measured at 513 nm. The concentration of elastin was calculated from a linear regression graph of elastin standard.

### Wound healing assay

HDFn cells cultured in DMEM supplemented with 10% fetal bovine serum and 1% antibiotic–antimycotic solution. Cells were maintained at 37 °C in a humidified incubator with 5% CO_2_. For the wound-healing assay, HDFn cells were seeded at a density 2 × 10^5^ cells/well in 24-well plates and incubated for 24 h to form a confluent monolayer. A uniform scratch was created across each well using a Culture-Insert 2 Well 24 (ibidi GmbH, Germany). Thereafter, the cells were treated with MPTR extract (0.125, 0.25, and 0.5 mg/mL). Untreated cells were used as the negative control. Cell migration was monitored at 0, 24, and 48 h under a microscope at a magnification of ×10. The wound area was measured using Zeiss Zen (ZEN 2.6, blue edition; Carl Zeiss, Oberkochen, Germany). The data are presented as the percentage of the wound area relative to the condition at 0 h. Results are expressed as the percentage of wound area compared to the baseline at 0 h.

### Statistical analysis

All statistical analyses were performed using SPSS 28 software program (IBM, Armonk, NY, USA). Experimental data are expressed as mean ± standard deviation (SD). Statistical analyses were conducted using a *t-test* for comparisons between two groups or a one-way analysis of variance (ANOVA) followed by Fisher’s Least Significant Difference (LSD) post hoc test for comparisons among more than two groups. A *p < 0.05* was considered indicative of statistical significance.

## Results

### Characteristics of M. purpureus in cultures with and without 1% Tubtim chumphae broken rice at 3 and 7 days

**Figure 1.**
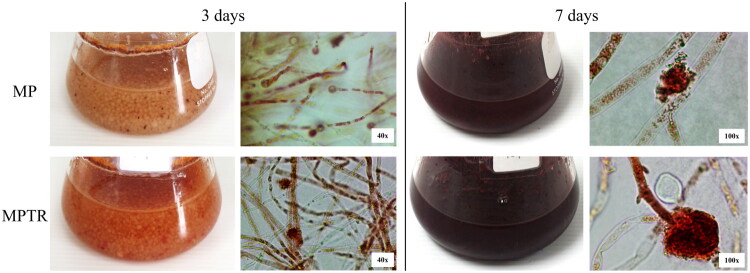
Characteristics of *Monascus purpureus* cultivated in potato dextrose broth (MP) and *M. purpureus* cultivated in potato dextrose broth supplemented with 1% Tubtim chumphae broken rice (MPTR) at 3 and 7 days, observed under a light microscope at ×40 and ×100 magnification. Mycelial morphology containing *Monascus* pigment derived from the culture media at 7 days, observed under a light microscope at ×100 magnification.

In this study, *M. purpureus* was cultivated in PDB (MP) and PDB containing 1% Tubtim chumphae broken rice (MPTR) in 250 mL flasks for 3 and 7 days. After 3 days, MP and MPTR exhibited orange and reddish-orange hues, respectively (Figure 1). However, both the MP and MPTR cultures produced reddish-orange pigments within the cells and medium after 7 days. Microscopic examination revealed the formation of 2–8 ascospores per ascus. These sexually produced spores develop within the ascus, and the asci are contained within an ovoid to globose ascocarp that exhibits a reddish brown to orange hue (Figure 1). Additionally, MP and MPTR were cultured in a 5 L bioreactor for 7 days and extracted with ethanol. Pigment analysis further showed that MPTR exhibited greater intensities in yellow, orange, and red pigments than MP, with the largest relative increase observed in the red (Table 1). The yields of MP and MPTR extracts were 19.31 ± 1.02 and 22.81 ± 1.19%, respectively.

**Table 1.** Pigment content and color characteristics of MP and MPTR in a bioreactor after 7 days of cultivation.

	MP	MPTR
Yield of extract (%)	19.31 ± 1.02^b^	22.81 ± 1.19^a^
Pigment content (AU/mL)		
Yellow	1.81 ± 0.004^b^	2.07 ± 0.007^a^
Orange	0.84 ± 0.001^b^	0.89 ± 0.004^a^
Red	0.73 ± 0.001^b^	0.97 ± 0.030^a^

Different letters a and b in the same row indicate significant differences (*p* < 0.05).

### Bioactive compounds of MP and MPTR extracts

LC–MS/IT-TOF analysis was performed to determine the key compounds in the pigments. As shown in Table 2. Among the six pigment compounds detected in MPTR extract, four compounds (rubropunctamine, monascorubramine, monascopyridine B, and monasfluor A) were responsible for the red color, and two compounds (monascin and ankaflavin) contributed to the yellow color. Although MP extract contained five compounds identified in MPTR extract, it lacked monasfluor A (Table 2). Although *M. purpureus* was cultured under neutral and alkaline conditions, citrinin was detected in both MP and MPTR extracts (Table 2).

**Table 2.** Compound identified in MP and MPTR extracts after 7 days of cultivation in a bioreactor.

No.	Compounds	Chemical	M.W.	Precursor ion (m/z)	Color
MP					
1.	Rubropunctamine	C_21_H_23_NO_4_	353.1627	354.17	Red
2.	Monascin	C_21_H_26_O_5_	358.178	360.19	Yellow
3.	Monascorubramine	C_23_H_27_NO_4_	381.194	382.20	Red
4.	Monascopyridine B	C_23_H_29_NO_4_	383.2097	384.12	Red
5.	Ankaflavin	C_23_H_30_O_5_	386.2093	387.22	Yellow
6.	Citrinin	C_13_H_14_O_5_	250.25	252.07	
MPTR					
1.	Rubropunctamine	C_21_H_23_NO_4_	353.1627	354.17	Red
2.	Monasfluor A	C_22_H_26_O_4_	354.18	357.17	Red
3.	Monascin	C_21_H_26_O_5_	358.178	359.19	Yellow
4.	Monascorubramine	C_23_H_27_NO_4_	381.194	382.20	Red
5.	Monascopyridine B	C_23_H_29_NO_4_	383.2097	384.12	Red
6.	Ankaflavin	C_23_H_30_O_5_	386.2093	387.22	Yellow
7.	Citrinin	C_13_H_14_O_5_	250.25	252.07	

### Antioxidant activity of MP and MPTR extracts

As shown in Table 3. MPTR extract exhibited significantly higher antioxidant activity than MP extract in both the DPPH^•^ and ABTS^•+^ assays. Notably, the IC_50_ values of MPTR and MP extracts against DPPH radicals were 0.88 and 1.25 mg/mL, respectively, confirming the superior antioxidant effect of MPTR extract. Similarly, MPTR extract demonstrated a superior inhibitory effect against ABTS^•+^ radicals than MP extract. Additionally, the antioxidant capacities of MP and MPTR extract were evaluated using FRAP assay. Consistent with the results of the DPPH^•^ and ABTS^•+^ assays, MPTR extract exhibited a significantly higher antioxidant activity (2.29 mg TE/g extract) than MP extract (1.33 mg TE/g extract).

**Table 3.** Antioxidant, anti-collagenase, and anti-elastase activities of MP and MPTR extracts derived from the bioreactor cultivation.

Sample	IC_50_ (mg/mL)		mg TE/g extract	IC_50_ (mg/mL)	
	DPPH^•^	ABTS^•+^	FRAP	Anti-collagenase	Anti-elastase
MP	1.25 ± 0.18^a^	1.01 ± 0.12^a^	1.33 ± 0.11^b^	1.79 ± 0.09^a^	1.89 ± 0.04^a^
MPTR	0.88 ± 0.22^b^	0.79 ± 0.16^b^	2.29 ± 0.09^a^	1.21 ± 0.03^b^	1.01 ± 0.10^b^
L-ascorbic acid	0.26 ± 0.01^c^	0.05 ± 0.02^c^	–	–	–
EGCG	–	–	–	0.09 ± 0.01^c^	0.07 ± 0.03^c^

Different letters in the same column indicate significant differences between samples (*p* < 0.05).

### Anti-collagenase and anti-elastase activities of MP and MPTR extracts

In this study, we compared the anti-collagenase and anti-elastase activities of the MP and MPTR extracts with those of EGCG, a well-known antioxidant. As shown in Table 3, EGCG showed superior inhibitory effects than MRTR and MP extracts, with IC_50_ values of 0.09 ± 0.01 and 0.07 ± 0.03 mg/mL against collagenase and elastase, respectively. However, MPTR extract exhibited stronger inhibitory effects than MP extract, with IC_50_ values of 1.21 ± 0.03 and 1.01 ± 0.10 mg/mL against collagenase and elastase, respectively. For the inhibitory activity of MP extract against collagenase and elastase, IC_50_ values were 1.79 ± 0.09 and 1.89 ± 0.04 mg/mL, respectively. This study found that MPTR extract from *M. purpureus* TISTR 3615 fermented with broken rice exhibited not only antioxidant properties but also collagenase and elastase inhibitory activities.

### Cytotoxicity of MPTR extract on HDFn

**Figure 2.**
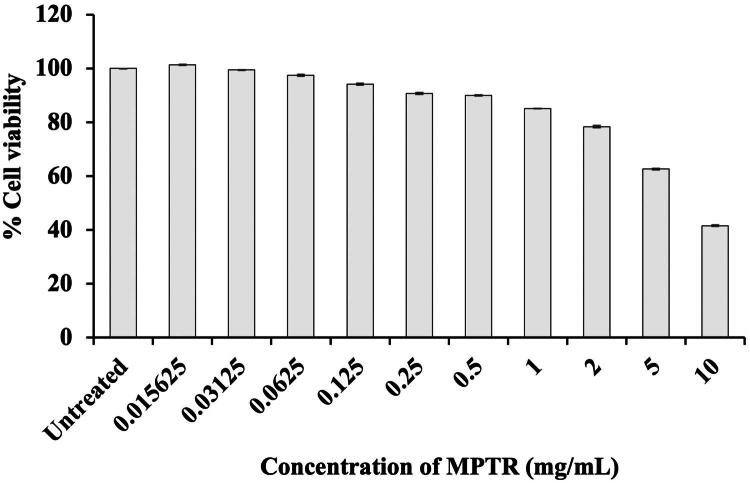
Viability of human dermal fibroblasts (HDFn) treated with *Monascus purpureus* cultivated in potato dextrose broth supplemented with 1% Tubtim chumphae broken rice (MPTR). Percentage cell viability is expressed as mean ± standard deviation (SD) of values from three independent experiments.

WST-1 assay was performed to determine the safe concentration of MPTR extract for human skin. HDFn survival of 90% or above indicated a safe concentration. Notably, the viability of HDFn treated with various concentrations of MPTR extract (0.015–0.5 mg/mL) ranged from 90.01 ± 0.07 to 101.31 ± 0.04%, indicating no cytotoxic effect on HDFn (Figure 2). However, cell viability decreased significantly at concentrations > 0.5 mg/mL, suggesting that cytotoxicity was concentration-dependent.

### MPTR extract promotes cellular collagen type I and elastin production

#### Cellular collagen type I production

**Figure 3.**
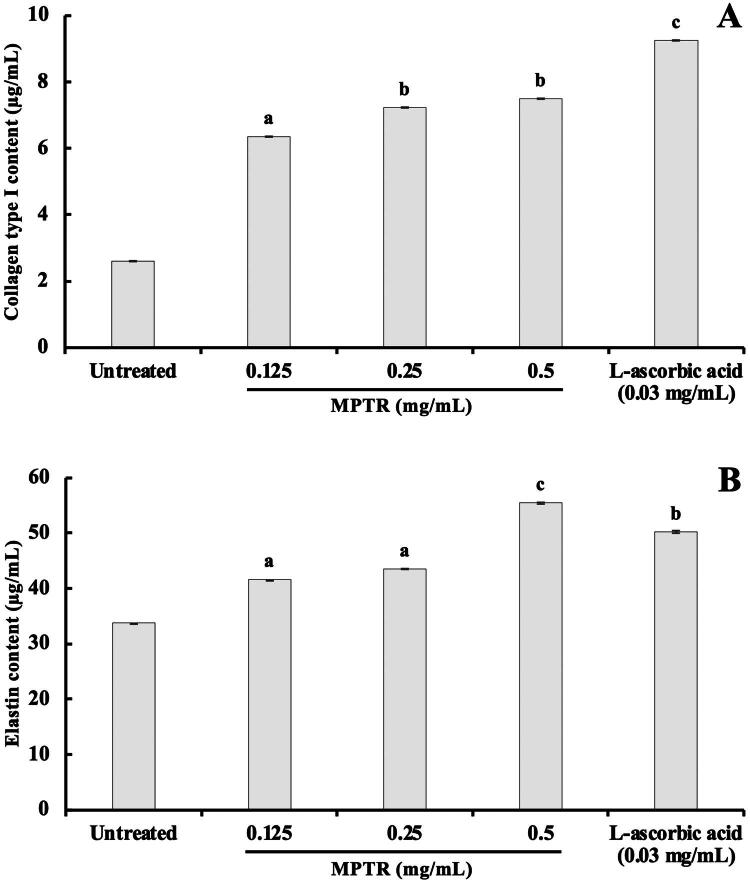
(A) Type I collagen content and (B) elastin content in human dermal fibroblasts (HDFn) following treatment with various concentrations (0.125, 0.25 and 0.5 mg/mL) of *Monascus purpureus* cultivated in potato dextrose broth supplemented with 1% Tubtim chumphae broken rice (MPTR) or L-ascorbic acid (0.03 mg/mL). Data are means ± SD from three independent experiments. Superscript letters ^(a,b,c)^ indicate significant difference at comparison with untreated (*p < 0.05*). Different superscript letters ^(a,b,c)^ indicate significant different among difference conditions.

To examine the effect of MPTR extract on cellular collagen production, HDFn were treated with various concentrations of MPTR extract (0.125, 0.25 and 0.5 mg/mL) or 0.03 mg/mL of L-ascorbic acid (positive control) for 24 h. (Figure 3(A)). Treatment with 0.5 mg/mL of MPTR extract significantly increased collagen production (collagen quantity of 7.23 ± 0.001 µg/mL) compared with untreated cells (collagen quantity of 2.61 ± 0.013 µg/mL). Compared with MPTR extract, L-ascorbic acid exhibited a higher collagen-stimulating effect, increasing collagen production to 9.25 ± 0.001 µg/mL.

#### Cellular elastin production

To investigate the effect of MPTR extract on elastic production, HDFn were treated with various concentrations of MPTR (0.125, 0.25, and 0.500 mg/mL) or 0.03 mg/mL of L-ascorbic acid (positive control) for 24 h (Figure 3(B)). Results showed that 0.5 mg/mL of MPTR significantly induced elastin production (elastin quantity equivalent to 55.39 ± 0.002 µg/mL), compared with untreated cells (elastin quantity equivalent to 33.71 ± 0.001 µg/mL). Additionally, MPTR increased elastin production higher than L-ascorbic acid (elastin quantity equivalent to 50.22 ± 0.001 µg/mL).

This is the first finding to present that *Monascus* extract is able to promote both collagen type I and elastin production in HDFn. However, the mechanisms underlying MPTR-induced production of type I collagen and elastin are interesting for further investigation.

### Wound healing effect of MPTR extract

**Figure 4.**
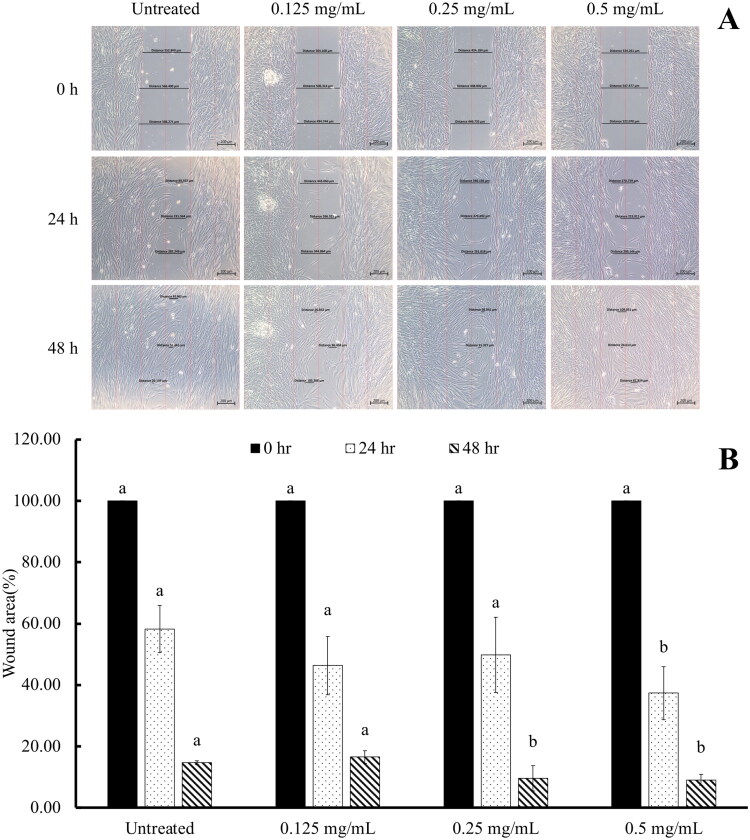
(A) Morphology of wound scratch in human dermal fibroblasts (HDFn) in the absence or presence of *Monascus* pigments (0.125, 0.25 and 0.5 mg/mL). (B) Percentage of wound closure in HDFn following treatment with or without *Monascus* pigment (MPTR; 0.12, 0.25 and 0.5 mg/mL). Data are means ± SD from three independent experiments. Superscript letters ^(a,b)^ indicate significant difference at *p < 0.05*. Superscript letter ^(a)^ indicates a significant difference when compared to cells at 0 h, whereas ^(b)^ indicates a significant difference when compared to untreated cells at *p < 0.05.*

To investigate the wound healing potential of MPTR, HDFn were treated with various concentrations of MPTR (0.125, 0.25, and 0.5 mg/mL) following injury, and the wound area was measured at 0, 24, and 48 h. MPTR treatment resulted in a progressive decrease in the wound area over time (Figure 4(A,B)), with the highest concentration (0.5 mg/mL) demonstrating the most significant healing effect. At 24 h, treatment with 0.5 mg/mL of MPTR significantly reduced the wound area to 37.36 ± 8.63%, compared with untreated cells (wound area 58.21 ± 7.64%). Treatment with 0.25 and 0.5 mg/mL of MPTR at 48 h, significantly decreased the wound area to 9.61 ± 4.14 and 9.10 ± 1.77%, respectively, compared to the untreated group (wound area 14.70 ± 0.68%). Additionally, treatment with the lowest concentration of MPTR (0.125 mg/mL) decreased the wound area to 16.53 ± 2.03% at 48 h.

## Discussion

The significant increase in yellow and red pigment production observed in this study can be attributed to the use of broken rice as a carbon source in the cultivation medium of *M. purpureus*. Rice, with its high glucose and soluble starch contents, serves as an excellent carbon source. Previous studies have shown that glucose and soluble starch are more effective than other carbon sources in promoting both growth and pigment synthesis (Lee et al. 2001; Singh et al. 2015). Additionally, rice contains nitrogen, which plays a critical role in modulating pigment production, influencing both cell growth and the quantity and quality of pigments produced by *M. purpureus* (Jung et al. 2003). The analysis of the secondary metabolites revealed that the vibrant hues (red, orange, or yellow) observed in *Monascus*-fermented substrates were attributed to a mixture of oligoketide pigments synthesized through the collaboration of polyketide and fatty acid synthases (Mostafa and Abbady 2014; Abel et al. 2023). Important substances in the extracts of MP and MPTR were found to contain five pigments (rubropunctamine, monascin, monascorubramine, monascopyridine B, and ankaflavin) and six pigments (rubropunctamine, monasfluor A, monascin, monascorubramine, monascopyridine B, and ankaflavin), respectively. Notably, rubropunctamine and monascorubramine are red pigment derived from azaphilone polyketides through the incorporation of amino groups, while ankaflavin and monascin are yellow azaphilones with strong antioxidant potential. Orange pigments, such as rubropunctatin and monascorubrin, are typically the lactone precursors of the corresponding red pigments, but were not detected as major compounds in the present LC-MS profiles, likely due to conversion during fermentation (Gong et al. 2023). Overall, these findings indicated that the MPTR pigments exhibit more intense colors than MP, particularly in the yellow and red spectra. Remarkably, this study revealed that the use of 1% Tubtim chumphae broken rice induced the production of monasfluor A, a minor *Monascu*s polyketide pigment, which is commonly observed in *M. purpureus* grown on rice (Patakova 2013). Previous research has indicated that monasfluor A and monasfluor B are newly identified red pigments in *M. purpureus* (Mukherjee and Singh 2011). The identification of monasfluor A exclusively in MPTR is particularly noteworthy, as this rare fluorinated red pigment has been reported in *M. purpureus* grown on rice-based substrates and is associated with enhanced color stability and potential bioactivities (Patakova 2013; Mukherjee and Singh 2011). The compositional differences between MP and MPTR suggest that supplementation with Tubtim chumphae broken rice not only increases pigment yield but also alters the biosynthetic pathway, favoring the production of unique secondary metabolites. The presence of monasfluor A in MPTR extract may be related to the pH of the cultivation medium, which influences the production of pigments. At pH 7, the metabolic pathways of *Monascus* often shift toward increased production of pigments, such as *Monascus* pigments, and decreased production of citrinin (Orozco and Kilikian 2008). In our study, citrinin was also found in both MP and MPTR extracts. According to Orozco and Kilikian (2008), citrinin production by *M. purpureus* is significantly affected by the pH of the culture medium. Citrinin production tends to decrease under neutral and alkaline conditions because these conditions are less favorable for its biosynthesis. Citrinin synthesis is generally more active under slightly acidic conditions (pH 5–6). However, certain *Monascus* strains do not produce citrinin or citrinin-free domesticated strains (Wang et al. 2023), which may serve as promising alternatives in the future.

Bioactivities of MP and MPTR extracts, including antioxidant capacity, collagenase and elastase inhibitory activities, cytotoxicity, and elastin- and collagen-stimulating properties, and wound healing were studied. Antioxidants are essential for protecting the skin against oxidative damage caused by free radicals. We examined the antioxidant properties of MP and MPTR extracts colorants using the DPPH^•^ and ABTS^•+^ scavenging assays, and the results exhibited that MPTR extract exhibited significantly higher antioxidant activity than MP extract in both the DPPH^•^ and ABTS^•+^ assays. Consistent with previous findings (Amić et al. 2003), our study showed that *Monascus* extracts, particularly MPTR, possess strong antioxidant activity and effectively scavenge free radicals. Wu et al. (2021b) reported that yellow pigments produced by *Monascus* spp, such as ankaflavin, possessed antioxidant properties, with ankaflavin exhibiting stronger activity than monasfluor A. Importantly, the antioxidant activities of other pigments, such as rubropunctamine, monascin, and monascorubramine, have also been documented (Dhale et al. 2007; Yeh et al. 2012; Koli et al. 2019). Collectively, these pigments contribute to the overall antioxidant capacity of *Monascus* extract. Matrix metalloproteinases (MMPs), including collagenase (MMP-1) and elastase, degrade key structural proteins in the skin such as collagen and elastin, which are essential for skin integrity and elasticity (Philips et al. 2011; Thring et al. 2009; Tzaphlidou 2004; Pittet et al. 2014). Uncontrolled activity of these enzymes contributes to skin aging and wrinkle formation.

In this study, *Monascus* extracts, particularly MPTR extract, effectively inhibited both collagenase and elastase enzymes. One previous report presented that extracts from *M. pilosus* KCCM 60084 fermented with soybeans exhibited antioxidant properties and inhibitory activities against skin aging-related enzymes, including tyrosinase, hyaluronidase, and elastase (Jin and Pyo 2017). Additionally, *Monascus* pigments, classified as azaphilones, are associated with biological activities, including enzyme inhibition for anti-obesity, hyperlipidemia, and hyperglycemia (Liu et al. 2018). Although synergistic interactions between the components in MPTR cannot be ruled out, the results suggest that *Monascus*-derived metabolites are likely responsible for the observed effects, displaying inhibitory activity against skin aging-related enzymes and possessing antioxidant properties. This is the first report to present that *Monascus* extract is able to promote both collagen type I and elastin production in HDFn. However, the mechanisms underlying MPTR-induced production of type I collagen and elastin warrant further investigation. Azaphilone pigments such as monascin, ankaflavin, rubropunctamine, and monascorubramine exert their anti-aging effects primarily through two pathways: (i) inhibition of matrix metalloproteinases (MMPs), particularly collagenase (MMP-1) and elastase, thereby preventing degradation of extracellular matrix (ECM) proteins, and (ii) activation of the transforming growth factor-beta (TGF-β)/Smad signaling pathway, which enhances fibroblast activity and stimulates the synthesis of collagen type I and elastin fibers. Furthermore, these pigments possess potent antioxidant activity that scavenges reactive oxygen species (ROS), reducing oxidative stress-induced activation of MMPs. By downregulating pro-inflammatory mediators such as IL-6 and TNF-α, azaphilones indirectly create a favorable microenvironment for ECM remodeling and skin regeneration (Van Doren 2015; Chen et al. 2017; Wu et al. 2021a; Husakova and Patakova 2025). In several studies of other fungi, Ruggeri et al. (2023) demonstrated that the mycelia of *Ganoderma lucidum* and *Pleurotus ostreatus* are biocompatible and enhance the expression of collagen I gene. Furthermore, the extract from the medium used to cultivate *Aspergillus chevalieri* TM2-S6, isolated from the sponge *Axinella*, contains tetrahydroauroglaucin and flavoglaucin, which play a crucial role in promoting cell viability and protecting human skin from oxidative stress *in vitro* (Letsiou et al. 2020). However, most studies have reported that extracts from molds exhibiting collagenase and elastase inhibition, along with collagen synthesis, are predominantly found in mushrooms such as *Tricholoma matsutake*, *Agaricus blazei* Peck, *Tremella fuciformis*, *Cordyceps cicadae*, and *Volvariella volvacea*, but no report in *Monascus* spp. and other fungi (Shao et al. 2023; Hu et al. 2024; Paterska et al. 2024). Considering fungal vital roles in skin health, stimulation of collagen and elastin production is a key target in anti-aging therapies. In the healing effect of MPTR extract on wounds, the results suggest that MPTR promotes wound healing *in vitro* in a concentration-dependent manner. Notably, the enhanced wound healing effect of MPTR may be attributed to its bioactive compounds, including *Monascus*-derived pigments and secondary metabolites, which are known for their anti-inflammatory and tissue regenerative properties (Husakova and Patakova 2025). Similarly, previous studies have reported the wound-healing effects of fungal-derived compounds (Arslan et al. 2024). Collectively, *Monascus* pigments and their bioactivities are highlighted to support the potential of MPTR extract as a natural therapeutic agent for skin anti-aging and regeneration.

## Conclusions

Our study shows that MPTR extract from *M. purpureus* cultivated in PDB supplemented with 1% broken rice exerts a superior antioxidant effect and ability to inhibit collagenase and elastase than MP extract from *M. purpureus* cultivated in PDB. Interestingly, MPTR extract promotes collagen and elastin production and possesses wound healing effects in HDFn. Importantly, the bioactive properties of MPTR extract are attributed to the presence of *Monascus*-derived pigments and secondary metabolites. Collectively, these findings highlight the promising applications of MPTR extract in the development of natural-based cosmetic and therapeutic products, particularly for skin regeneration and anti-aging treatments.

## Data Availability

This study does not have associated data.
